# Complete Genome Sequences of Three Ornithobacterium rhinotracheale Strains from Avian Sources, Using Hybrid Nanopore-Illumina Assembly

**DOI:** 10.1128/mra.01059-22

**Published:** 2023-01-31

**Authors:** Amro Hashish, Timothy J. Johnson, Emily Smith, Dhiraj Chundru, Michele L. Williams, Nubia R. Macedo, Yuko Sato, Mostafa Ghanem, Mohamed El-Gazzar

**Affiliations:** a Department of Veterinary Diagnostic and Production Animal Medicine, College of Veterinary Medicine, Iowa State University, Ames, Iowa, USA; b Department of Veterinary and Biomedical Sciences, College of Veterinary Medicine, University of Minnesota, St. Paul, Minnesota, USA; c Department of Veterinary Medicine, Virginia-Maryland College of Veterinary Medicine, Blacksburg, Virginia, USA; d National Laboratory for Veterinary Quality Control on Poultry Production, Animal Health Research Institute, Agriculture Research Center, Giza, Egypt; Loyola University Chicago

## Abstract

Ornithobacterium rhinotracheale has been associated with respiratory disease in poultry, particularly turkeys, leading to significant economic losses. However, O. rhinotracheale is poorly studied, and a very limited number of complete genomes are available. Here, we report the complete genome sequences of three O. rhinotracheale strains, generated using a Nanopore-Illumina hybrid assembly approach.

## ANNOUNCEMENT

Ornithobacterium rhinotracheale has emerged as an important bacterial pathogen for domestic poultry and wild birds (1–10); however, it is an understudied pathogen. Only seven complete genomes are available in GenBank (https://www.ncbi.nlm.nih.gov/genome/browse/#!/prokaryotes/10734/) to date (November 2022), three of which are from this study. Here, we report the complete genome sequences of three O. rhinotracheale strains, generated using a Nanopore-Illumina hybrid assembly approach (11). These complete genomes will facilitate better understanding of this pathogen.

Three O. rhinotracheale isolates were selected as a diverse group of isolates obtained from the laboratory of K. Nagaraja at the University of Minnesota. As described previously (12), bacterial reculture was performed on blood agar (with 5% sheep blood), and the cultures were incubated under microaerophilic conditions at 37°C for 48 h. Isolates were identified and confirmed as O. rhinotracheale using real-time PCR as described previously (13). DNA extraction was performed using a MagMAX pathogen RNA/DNA kit (Thermo Fisher Scientific, Waltham, MA, USA) on a Kingfisher Flex instrument (Thermo Fisher Scientific). Extracted DNA was used for sequencing library preparation with an Illumina DNA preparation-tagmentation kit with IDT for Illumina DNA/RNA unique dual (UD) indexes, set A (Illumina, USA). The sequencing was performed using the Illumina MiSeq system (v3 reagent kit, with 2 × 300-bp paired-end reads).

DNA extraction for Nanopore sequencing was performed with the Nanobind CBB Big DNA kit (Circulomics, Baltimore, MD, USA) using the Gram-negative bacteria high molecular weight DNA extraction protocol. Nanopore libraries were prepared by multiplexing DNA extracts from the three isolates using the SQK-LSK109 and EXP-NBD104 kits (Oxford Nanopore Technologies, UK) according to the manufacturer's protocol. Barcodes 1, 3, and 7 from the EXP-NBD104 kit were used.

Raw reads from Illumina sequencing were first trimmed for quality and sequencing adapters using Trimmomatic v0.33 (14) with default parameters. NanoFilt v2.8.0 (15) was used with default parameters to quality filter Nanopore reads and filter out sequences <1,000 bp in length. Hybrid assemblies were performed using Unicycler v0.5.0 (16) with default parameters. All genomes were annotated using the NCBI Prokaryotic Genome Annotation Pipeline (PGAP) v6.1 (17) as part of the RefSeq prokaryotic genome annotation project (BioProject accession number PRJNA224116). Reads generated with each sequencing platform, assembly statistics, annotation of the closed genomes, sequence typing, average nucleotide identity (ANI) values, and GenBank accession numbers are presented in Table 1. The hybrid assembly resulted in the formation of complete, closed circular genomes for the three isolates. However, the number of contigs for K223 is two due to the presence of a plasmid, which resulted in one closed contig for the chromosome and one closed contig for the plasmid.

**TABLE 1:** Sequencing, annotation, and typing results for the sequenced strains

Parameter^*a*^	Data for O. rhinotracheale strain:		
	A171	K223	97
Raw sequence reads			
Illumina paired-end read length (nucleotides)	300	300	300
No. of Illumina reads used	99,581	130,665	50,152
Avg Illumina coverage (×)	381	484	62
No. of Nanopore reads	27,383	21,287	6,593
Avg Nanopore coverage (×)	97.08	75.18	97.12
Nanopore read *N*_50_ (bp)	15,541	17,237	2,188
Assembly statistics for closed genomes			
No. of contigs	1	2	1
Total chromosome length (bp)	2,409,995	2,407,355	2,418,866
Plasmid^*b*^	Absent	Present	Absent
Total plasmid length (bp)	NA	14,785	NA
GC content (%)	37.45	37.37	37.43
Hybrid assembly *N*_50_ (bp)	2,409,995	2,407,355	2,418,866
Annotation results			
No. of CDSs (protein)	2,237	2,289	2,247
No. of noncoding RNAs	3	3	3
No. of tRNAs	43	43	43
No. of rRNAs	9	9	9
No. of CDSs (without protein)	26	29	29
No. of CRISPR arrays	1	1	1
No. of hypothetical proteins	541	563	568
No. of proteins with functional assignments	1,697	1,726	1,679
Serotype from agar gel precipitation test	A	K	Unknown
ST from O. rhinotracheale MLST^*c*^	ST-1	NA (too divergent to conclude ST)	ST-1
ANIb (%) vs O. rhinotracheale type strain DSM 15997 (accession no. CP003283.1)^*d*^	99.9	89.49	99.86
GenBank accession no.			
BioSample accession no.	SAMN27124716	SAMN27139539	SAMN27150225
BioProject accession no.	PRJNA821850	PRJNA821858	PRJNA821870
Complete genome assembly accession no.			
Chromosome	CP094844	CP094846	CP094845
Plasmid		CP094847	

a CDS, coding sequence; ST, sequence type; MLST, multilocus sequence typing; ANI, average nucleotide identity; NA, not applicable.

b The presence or absence of plasmids in the genome sequences was determined using the Plasmid database (19) via PLSDB (https://ccb-microbe.cs.uni-saarland.de/plsdb).

c MLST was performed using the O. rhinotracheale MLST scheme (20) available via PubMLST (21) (https://pubmlst.org/organisms/ornithobacterium-rhinotracheale).

d ANIb are ANI values based on BLAST+ calculated via the JSpeciesWS online service (22) (https://jspecies.ribohost.com/jspeciesws/#home).

It is noteworthy (as shown in Table 1) that the ANI values for two genomes (A171 and 97) versus the O. rhinotracheale type strain DSM 15997 (accession number CP003283.1) were above the species cutoff value of 95 to 96%, while the ANI value for genome K223 was below the species cutoff value (89.49%). These findings support the suggestion by Alispahic et al. (18) that some O. rhinotracheale serotypes belong to a different bacterial species.

### Data availability.

The genomes are available in GenBank under the accession numbers shown in Table 1.
